# Elevated levels of anti inflammatory IL-10 and pro inflammatory IL-17 in malignant pleural effusions

**DOI:** 10.1186/1749-8090-7-104

**Published:** 2012-10-04

**Authors:** Michail Klimatsidas, Kyriakos Anastasiadis, Christophoros Foroulis, Paschalis Tossios, Alexandros Bisiklis, Christos Papakonstantinou, Kyriakos Rammos

**Affiliations:** 1 Thoracic Surgery Department, 424 Military Hospital of Greece, Thessaloniki, Greece; 2 Biomedical Department, 212 Military Hospital of Greece, Xanthi, Greece; 3 Cardiothoracic Surgery Department, AHEPA University Hospital of Thessaloniki, Thessaloniki, Greece

## Abstract

**Background:**

Pleural effusions can be caused by highly different underlying diseases and are characterized by complex interactions of various local and circulating cells as well as numerous soluble parameters like interleukins (IL). Knowledge of this complex network can be helpful in order to make the differential diagnosis in known malignant pleural effusions and understand the underlying immunochemistry of each disease or condition.

**Methods:**

We investigated immunoreactive concentrations of Interleukin 10 (IL-10) and Interleukin 17 (IL-17) in malignant pleural effusions and peripheral blood from patients with bronchial carcinomas and other carcinomas, excluding other conditions such as congestive heart failure (CHF) and pneumonias in twenty four (24) patients (9 men/15 women), 37-74 years (mean:61) with already diagnosed malignant pleural effusions applying the ELISA method.

**Results:**

The SPSS 15 program for Windows was used. Quantitative analysis showed high concentrations of IL-10 and IL-17 in pleural fluid and blood. Even though IL-17 levels -both blood and pleural- were lower than IL-10’s, statistical correlation between blood and pleural concentations was proven, confirming once more the systematic action of these cytokines. At the same time high IL-17 levels in malignant effusions shows maybe a new perspective in understanding the pathophysiology of malignant pleural effusions.

**Conclusions:**

Our results confirm the pathogenetic role of these cytokines in malignant pleural effusions combining for the first time a pro- and an anti- inflammatory cytokine. The observation that IL-17 is elevated in malignant pleural effusions may give a new meaning in Virchow’s remarks 100 years ago. Larger number of patients is needed to confirm our hypothesis.

## Background

Pleural effusions are common clinical problems, occuring in more than 1 million patients each year. Up to 22% of these effusions are caused by malignant disease and more than 100.000 malignant effusions require treatment annually. [1] Affected patients with advanced neoplastic disease experience considerable morbidity.

Pleural effusion results from a derangement in the normal physiology such that there is increased production of pleural fluid or a change in its composition with or without a reduction in the absorption of the fluid. It can result from primary or secondary tumors of the pleura, with seeding of the intrapleural space and lymphatic obstruction. Free-floating tumor cells block absorption of fluid and produce vasoactive substances that in turn increase the production and block the absorption of intrapleural protein and fluid.

In adults, 95% of neoplastic pleural effusions arise from a metastatic source, with lung and breast carcinoma accounting for 75% of all cases. Other common causes are lymphoma, gastric cancer, and ovarian cancer. About half of patients with breast cancer develop a pleural effusion within their lifetime, compared with one fourth of patients with lung cancer and one third of patients with lymphoma.

Most pediatric effusions, on the other hand, are benign. If malignant, lymphomas or leukemias account for half, with the remainder a mix of tumors such as neuroblastoma, Wilms’ tumor, and germ cell neoplasms.

Light’s criteria [2] are the golden standard regarding the diagnosis of pleural effusion being a transudate or an exudate. In many occasions though, these criteria are not sufficient in posing a definite diagnosis or the can even misclass an effusion e.g. when a patient is on diuretics [3]. Pubmed search will reveal numerous attempts to provide markers or measurements that will help us provide a definite diagnosis or even a prognosis from a pleural effusion.

Both immunocompetent cells and tumour cells secrete cytokines, which have an important role in mediating (down regulating and up regulating) cellular and humoral activity [1].

## Patients and methods

72 consecutive patients that had chest drain insertion or pleural tap in the Thoracic surgery department were included in the study over a period of 6 months.

All patients were consented for participating in this study.

We measured the levels of Interleukins 10 and 17 in patients with malignant pleural effusions. Patients were included only if the effusion was an exudate and a definite cytologic diagnosis of malignant cells in the pleural fluid, was made.

Exclusion criteria included absence of malignant cells in the cytology report, a definite diagnosis of effusion of other origin (e.g. cardiac failure), pleural fluid was a transudate or patients were under the treatment of chemotherapy or corticosteroid therapy at the time of the chest drain insertion or in the last three months.

All these conditions were excluded so as to minimize the influence of them in the results.

24 patients fulfilled the above mentioned criteria 9 males and 15 females, aged from 37 to 74 years (mean = 60) (Table 1).

**Table 1:** Descriptive statistics

	**N**	**Minimum**	**Maximum**	**Mean**	**Std. Deviation**
IL- 17 B	24	18.258	31.605	23.63281	3.553243
IL-10 B	24	.00	112.43	13.8533	28.72131
IL-17 P	24	21.009	33.020	24.97380	3.595384
IL-10 P	24	1.75	229.83	60.1112	59.13965
WBC	24	2.3	30.6	10.296	5.5273
RBC	24	3.16	5.17	3.9788	.53680
HCT	24	28.4	46.5	36.192	4.7277
PLT	24	87	658	283.71	117.411
HB	24	9	16	12.02	1.675
GR%	24	43.5	93.8	71.912	10.7272
MONO%	24	.8	30.6	9.846	6.6490
LYMPH%	24	1.4	32.1	17.308	7.5535
AGE	24	37	74	60.42	10.270
GENDER	24	1	2	1.63	.495
AGEgroup	24	1	3	2.50	.659
primary1	24	1	2	1.63	.495
TYPE_3_UN	24	1	3	2.04	.624
chemo1yes	24	0	1	.58	.504
Valid N (listwise)	24				

*IL-17B* Interleukin 17 levels in Blood, *IL-10B* Interleukin 10 levels in Blood, *IL-17P* Interleukin 17 levels in Pleural Fluid, *IL-10P* Interleukin 10 levels in Pleural Fluid, *WBC* White Blood Cells Count, *RBC* Red Blood Cells Count, *HCT* Heamatocrit, *PLT* Platelets, *HB* Heamoglobulin, *GR%* Granulocytes percentage, *MONO%* Monocytes percentage, *LYMPH%* Lymphocytes percentage, *AGE* Age, *GENDER* Gender, *AGEgroup* 1 = age 1 to 39, 2 = 40 to 59, 3 = 60 and above, *Primary1* 1 = Lung cancer, 2 = other, *TYPE_3_UN* 1 = NSCLC,SCLC,AdenoCa Lung/2 = other malignancy/3 = Unknown origin of malignancy, *chemo1yes* chemotherapy done = 1.

Pleural effusions were collected during the first diagnostic or therapeutic thoracocentesis. Patients were consented before the chest drain insertion for the research project.

The pleural fluid was placed in tubes containing ethylene diamine tetra-acetate (EDTA) to a final concentration of 1 mg/ml. A separate portion was placed in plain tubes for determination of LDH and total protein. Blood was withdrawn from peripheral vein and collected in tubes containing EDTA (final concentration 1 mg/ml) or in plain tubes. The supernatants and cell pellets were separated by centrifugation (600 g, 30 min, 4°C). Plasma, serum and pleural effusion supernatants were aliquotted and stored at −70°C for later analysis. All the collected samples were tested within 1 month after collection. Positive and negative controls were included in the assay.

Immunoreactive concentrations of IL-10 and IL-17 were determined in effusion fluid and blood plasma samples utilizing commercially available ELISA assays [TiterZyme® EIA, ELISYS QUATRO, (IL-10)], [Quantikine, R&D Systems GmbH, Wiesbaden, FRG (IL-17)]. The lower detection limits were: IL-10 [3.75 pg/ml]; IL-17 [15 pg/ml].

The cytokine level in the samples was determined by ELISA method that used the quantitative sandwich enzyme immunoassay technique.

### Statistical analysis

Descriptive statistics were applied in every result. All data are presented as mean + − standard error.

All variables were tested for normality of their distribution by the Shapiro test. For non-parametric quantitative variables, data were subjected to Spearman analysis and tested with Mann- Whitney *U* test. For parametric variables, Pearson test was used for correlations. We have used the SPSS 15 statistical package for Windows. Statistical Significance was set to p <0.05.

## Results

The mean values of all the measurements are presented in Table 2. Interleukin’s 10 levels in blood ranged between 0–112.43 pg/ml, mean 13.85 pg/ml while Interleukin’s 17 blood levels were between 18.25 - 31.605 pg/ml mean 23.63 pg/ml. Il-17 pleural levels were between 21.00 pg/ml and 33.02 pg/ml mean 24.97 pg/ml while IL-10 P was 1.75 -229.83 pg/ml with a mean 60.11 pg/ml.

**Table 2:** Concentrations of IL-10 and IL-17 in Pleural fluid and Blood of all the patients

	**IL-10P (pg/ml)**	**IL-10B (pg/ml)**	**IL-17P (pg/ml)**	**IL-17B (pg/ml)**	**TYPE**	**AGE**	**GENDER**
**Mean**	60.1	13.85	24.97	23.63	9 chest cavity primary	60.4	9 m/15f

*IL-10P* levels of IL-10 in pleural fluid, *IL-10B* levels of IL-10 in Blood, *IL-17P* levels of IL-17 in pleural fluid, *IL-17B* levels of IL-17 in Blood.

9 out of 24 of the patients (37,5%) had chest cavity as the primary site of malignancy, while all the rest were considered as metastatic. In total 5 patients had metastatic breast cancer, 2 had ovarian cancer, 1 neuroglioma, 1 lymphoma and 5 patients had metastatic pleural disease from unknown primary origin. All patients had a confirmed diagnosis with cytology analysis and Medical Research Council (MRC) marked dyspnea 4 and 5 [4]. Quantitative analysis showed high concentrations of IL-10 and IL-17 in pleural fluid and blood. Interestingly enough, levels of IL-17P were strongly correlated with the levels of IL-17B (p = 0,000) (Table 3). Levels of IL-10B, IL-17B and IL-17P didn’t differ significantly when comparing men and women but levels IL-10P did differ (p = 0,037). Levels of IL-17B were much higher in patients who had as primary the chest cavity (p = 0,015) (Table 4).

**Table 3:** Correlations

	**Correlations**			
	**IL −10 B (IL-10 Concentration in Blood)**	**IL −10 P (IL-10 Concentration in Pleural fluid)**	**IL −17 B (IL-17 Concentration in Blood)**	**IL −17 P (IL-17 Concentration in Pleural fluid)**
IL −10 B (IL-10 Concentration in Blood)	-	P = 0,468	**P = 0,002**	P = 0,166
IL −10 P (IL-10 Concentration in Pleural Fluid)	P = 0,468	-	P = 0,393	P = 0,224
IL −17 B (IL-17 Concentration in Blood)	P = 0,002	P = 0,393	-	**P = 0,000**
IL −17 P (IL-17 Concentration in Pleural Fluid)	P = 0,166	P = 0,224	P = 0,000	-
WBC	P = 0,508	P = 0,888	P = 0,860	P = 0,877
RBC	P = 0,896	P = 0,951	P = 0,761	P = 0,764
HCT	P = 0,615	P = 0,992	P = 0,405	P = 0,433
PLT	P = 0,438	P = 0,847	P = 0,308	**P = 0,004**
HB	P = 0,616	P = 0,699	P = 0,558	P = 0,410
GRANULOCYTES%	P = 0,285	P = 0,722	P = 0,559	P = 0,308
MONOCYTES%	P = 0,660	P = 0,449	P = 0,839	P = 0,433
LYMPHOCYTES%	P = 0,185	P = 0,942	P = 0,404	P = 0,298

**Table 4:** Testing Interleukins levels with age (var = AGEGroups), primary lung cancer or not (var = primary1yes), had chemotherapy or not in the last 6 months (chemo1yes)

	**IL −10 B (IL-10 Concentration in Blood)**	**IL −10 P (IL-10 Concentration in Pleural fluid)**	**IL −17 B (IL-17 Concentration in Blood)**	**IL −17 P (IL-17 Concentration in Pleural fluid)**
**AGE***(ANOVA)*	P = 0,251	P = 0,969	P = 0,883	P = 0,067*
**Primary or Metastatic***(t-test)*	P = 0,112	P = 0,114	**P = 0,015**	P = 0,467
**Chemotherapy or not***(t-test)*	P = 0,127	P = 0,237	P = 0,133	P = 0,326
**Type of malignancy**	**P = 0,002**	P = 0,404	P = 0,034	P = 0,593
**SEX***(t-test)*	P = 0,517	**P = 0,037**	P = 0,349	P = 0,773

*Was tested with multiple comparisons, no statistical significance reached.

**Three groups were used (var = Type_3_UN).

## Discussion

Management of malignant pleural effusions is a major challenge. Large numbers of various variables and parameters exist and are useful weapons in our armamentarium in the differential diagnosis of the origin in any pleural effusion.

Light’s criteria [2] are great in distinguishing the transudates from the exudates. But, despite the numerous bibliographic attempts they haven’t been prooven to be sufficient in providing definite diagnosis. They were proved to be not enough, not only in finding out when an effusion was a malignant one or not, but also in the differential diagnosis of the histopathology type regarding the primary tumor. As a result, dozens of markers have been tested all these years [1].

One of the latest additions in this differential diagnosis marathon are cytokines. The complexity of the tumor microenvironment is characterized by the presence of multiple immune effector cells and a network of soluble factors such as cytokines [5].

Cytokines are important signalling molecules in the immune system. The immune response to foreign antigens is based on the remarkable activities of a rather small number of lymphocytes that specifically recognise each antigen [6,7]. Cytokine levels are not just another tool in distinguishing the type of malignancy but also a very module in understanding the pathophysiology of malignant pleural effusions and finally providing the evidence needed in the design of new treatment approaches for example cytokine specifically designed antibodies [8][9]. Since 1994 [10] has been found that Bronhogenic Lung Cancer produces IL-10 and there were thoughts that this might be the reason that the host defense was suppressed. In 2000 De Vita et al. [11] showed that patients with progressed disease of Small Cell Lung Cancer had increased serum levels of IL-10 which, were not associated with survival. A seminal paper regarding the elevation of IL-10 in Thoracic malignancies is the paper of Chen et al. [12] where the elevation of IL-10 in malignant pleural effusions was shown to be statistically significant. Our data are in accordance with Chen et al. [12] findings. Since then, numerous attempts have been made to find out the exact role that IL-10 has to play in the prognosis of Lung cancer. Atwell et al. [4] showed that there is a balance between the different cytokines excreted in patients with a thoracic malignancy. In that concept, findings that low levels of IL-10 show bad prognosis [13] could not be consistent. This led Hatanaka et al. [14] to flag this matter up suggesting that all the results have to be examined very carefully as the in vivo conclusions of IL −10 action are very much different than ex vivo – including their own results. Li Zeng et al. [15] in 2009 stated that IL-10 promotes angiogenesis and resistance to apoptosis thus increasing the metastatic potential in Lung tumor cell lines. IL-10 has been regarded as an immune suppressant cytokine [5] based on experimental observations. Surprisingly it is thought to have anticancer potentials [16]. This anticancer activity of IL-10 both in animal models and human observations was thought to be a cooperation with other factors. Experiments showed that IL-10 administration of doses up to 25 ug/kg had no or very little toxicity and proinflammatory properties. Taking all these into consideration we can conclude that IL-10 actions are multi-factorial. There are differences in vitro when compared to in vivo action as well as differences regarding many other coefficients like disease stage or type of malignancy. Human body’s response is not a standardized one when it comes to the disease and shares a lots of common between inflammation and wound healing. It was suggested from the early 19^th^ century - by Rudolf Virchow in 1963- that cancer was linked to inflammation [17]. He noted leucocytes in neoplastic tissues and made the assumption that there is a connection between inflammation and cancer. At the same time, he suggested that the “lymphoreticular infiltrate” indicated the site of the primary origin. In the last decade this theory is coming back to light, adding more and more scientific knowledge that chronic inflammation predisposes to tumor progressionand chemokines network is very likely that it contributes very much to this process [18]. More and more papers are trying to link inflammation and cancer again [17,19]. Nowadays, “subthreshold neoplastic states” caused by carcinogens as Peyton Rous first recognised from 1941 [20], are called “initiation” and “promotion” [21]. Cytokines might also help as our “biochemical microscope” in detecting and identifying malignancies.

Interleukin 17 (IL-17) is a cytokine secreted in large amounts exclusively by T-cells upon activation. Data available in vivo have supported both the proinflammatory and the hematopoietic activities of Il-17 e.g. in vivo intratracheal instillation of hiL-17 increased levels of rat macrophage inflammatory protein 2 (rMIP-2) in bronchoalveolar lavage (BAL) which selectively recruited neutrophils into rat airways [22]. No IL-17 has been detected in biological fluids under normal conditions. Biologically active IL-17 is spontaneously present in the supernatant from rheumatoid arthritis synovium pieces but not from osteoarthritis synovium, which contains a reduced T cell infiltrate [23]. The physiological functions of Il-17 remain to be established, in vitro and mouse in vivo data indicate that IL-17 might play a central role in several inflammatory diseases known to involve activated CD4+ T cells. IL-17 is now thought to have an active involvement in inducing and mediating proinflammatory responses. Overproduction of IL-17 has been associated with several chronic disease conditions, suggesting a role in the diseases [24]. This association of several members of this family with inflammation and the induction of other cytokines that impact the inflammatory response suggest that there may be therapeutic use in the blocking or regulating of the function of these proteins. Till now, to our knowledge, IL-17 has never been measured in malignant pleural effusions. The fact that IL-17 has been detected elevated in both pleural fluid and blood in patients with malignant pleural effusions shows a clear indication that inflammation and cancer are close -if not the same- pathways. Bibliography is enriched every day with papers suggesting the above, further research is needed.

## Conclusion

Elevated levels of inflammation related cytokines 10 and 17 in patients with malignant pleural effusions, is another possible indication that inflammation and cancer pathways are not only parallel ones but also at some points interconnecting ones also. They could be useful tools in understanding the pathophysiology of cancer but also a possible beginning in having a less invasive method - a so called "biochemical microscope"- in order to establish the diagnosis and the histology of a malignancy. Without any doubt further research is needed.

## Competing interests

The authors declare that they have no competing interests.

## Authors’ contributions

MK collected all the samples and data, helped in designing of the study and prepared the draft, KA, CF and PT supervised the study and made the neseccary corrections in the paper, AB made all the measurements and verified the data, CP and KR designed the study and helped in the initial funding and setup of the study. All authors read and approved the final manuscript.
